# The Hand and Wrist: AntImicrobials and Infection (HAWAII) trial

**DOI:** 10.1093/bjs/znad298

**Published:** 2023-09-27

**Authors:** Justin Conrad Rosen Wormald, Jeremy Rodrigues, Rinah Bheekharry, Nicholas Riley, Sarah Tucker, Dominic Furniss, Rebecca Dunlop, Robin Jones, Duncan Applebe, Kate Herbert, Daniel Prieto-Alhambra, Jonathan Cook, Matthew Lee Costa

**Affiliations:** Oxford Trauma and Emergency Care, Kadoorie Centre, Nuffield Department of Orthopaedics, Rheumatology and Musculoskeletal Sciences, University of Oxford, Oxford, UK; Warwick Clinical Trials Unit, Warwick Medical School, University of Warwick, Coventry, UK; Buckinghamshire Healthcare NHS Trust, Stoke Mandeville Hospital, Aylesbury, UK; Buckinghamshire Healthcare NHS Trust, Stoke Mandeville Hospital, Aylesbury, UK; Oxford University Healthcare NHS Foundation Trust, Oxford, UK; Oxford University Healthcare NHS Foundation Trust, Oxford, UK; Oxford University Healthcare NHS Foundation Trust, Oxford, UK; Botnar Research Centre, Nuffield Department of Orthopaedics, Rheumatology and Musculoskeletal Sciences (NDORMS), University of Oxford, Oxford, UK; Royal Cornwall Hospitals NHS Trust, Treliske, Truro, Cornwall, UK; Royal Cornwall Hospitals NHS Trust, Treliske, Truro, Cornwall, UK; Oxford Trauma and Emergency Care, Kadoorie Centre, Nuffield Department of Orthopaedics, Rheumatology and Musculoskeletal Sciences, University of Oxford, Oxford, UK; Oxford Trauma and Emergency Care, Kadoorie Centre, Nuffield Department of Orthopaedics, Rheumatology and Musculoskeletal Sciences, University of Oxford, Oxford, UK; Botnar Research Centre, Nuffield Department of Orthopaedics, Rheumatology and Musculoskeletal Sciences (NDORMS), University of Oxford, Oxford, UK; Botnar Research Centre, Nuffield Department of Orthopaedics, Rheumatology and Musculoskeletal Sciences (NDORMS), University of Oxford, Oxford, UK; Oxford Trauma and Emergency Care, Kadoorie Centre, Nuffield Department of Orthopaedics, Rheumatology and Musculoskeletal Sciences, University of Oxford, Oxford, UK

## Abstract

**Background:**

Hand trauma, comprising injuries to both the hand and wrist, affects over five million people per year in the NHS, resulting in 250 000 operations each year. Surgical site infection (SSI) following hand trauma surgery leads to significant morbidity. Triclosan-coated sutures may reduce SSI in major abdominal surgery but have never been tested in hand trauma. Feasibility needs to be ascertained before a definitive trial can be delivered in hand trauma.

**Methods:**

A multicentre feasibility RCT of antimicrobial sutures *versus* standard sutures involving adults undergoing surgery for hand trauma to evaluate feasibility for a definitive trial. Secondary objectives were incidence of SSI in both groups, hand function measured with patient-reported outcome measures, health-related quality of life and change in employment. Randomization was performed on a 1:1 basis, stratified by age of the patient and whether the injury was open or closed, using a secure, centralized, online randomization service. Participants were blinded to allocation.

**Results:**

116 participants were recruited and randomized (60 intervention, 56 control). Of 227 screened, most were eligible (89.5 per cent), and most who were approached agreed to be included in the study (84.7 per cent). Retention was low: 57.5 per cent at 30 days, 52 per cent at 90 days and 45.1 per cent at 6 months. Incidence of SSI was >20 per cent in both groups. Hand function deteriorated after injury but recovered to near pre-injury levels during the study period.

**Conclusions:**

Risk of SSI after hand trauma is high. A definitive RCT of antimicrobial sutures in hand trauma surgery is feasible, if retention is improved.

**Trial registration:**

ISRCTN10771059

## Teaser

Antimicrobial sutures might reduce wound infection following hand trauma surgery, but we do not know if we can test them in this population. We conducted a small-scale clinical trial of these sutures in hand trauma to ascertain if we could run definitive trials and how best to do so. We could easily recruit and randomize participants, but follow-up was challenging and will require careful planning in future studies.

Hand and wrist injuries, also known as ‘hand trauma’, account for one in five A&E attendances and affect five million people in the UK every year. This has a big impact on people’s lives, as the hand and wrist are important in daily life and for earning a living. Infection after surgery for hand injury causes major problems, including more antibiotics, more operations and readmission to hospital. Specially coated stitches, known as ‘antimicrobial stitches’, could reduce infection risk by killing bacteria in and around the wound. Preventing infection would improve recovery and return to normal life. To find out if antimicrobial stitches can be tested properly in hand trauma surgery, we performed a small-scale study comparing antimicrobial stitches with standard stitches in adults undergoing surgical operations for hand and wrist injuries. The study mainly aimed to assess the possibility of a larger, full-scale trial, but we also measured infection rates, hand function, quality of life and employment changes. The results suggest that a larger trial is possible if we can successfully follow people up after their operation.

## Background

Surgical site infection (SSI) in hand trauma is prevalent, occurring in at least 5–10 per cent of patients^1^. This exceeds the National Institute for Health and Care Excellence (NICE) estimate of 3–5 per cent for SSI across all surgical fields^2^. Despite this, interventions to reduce SSI risk have not been evaluated in hand trauma. This is important, as hand trauma predominantly affects a young, socioeconomically active population, who often require surgery for their injuries^3^. A number of antimicrobial interventions have been developed to reduce the risk of SSI across all surgery. Surgical suture material is commonly implicated in bacterial wound infection and is therefore a therapeutic target^4^. Antimicrobial sutures have been tested in a number of RCTs but have never been tested in hand trauma surgery. They are now increasingly available in the operating theatre across the UK. In a meta-analysis of 21 RCTs, sutures coated in Triclosan, an antimicrobial agent, reduced SSI in major abdominal and vascular surgery by 28 per cent^5^. Antimicrobial sutures are more expensive than standard sutures, although a recent economic evaluation of RCTs found antimicrobial sutures to be cost-effective in specific patient populations^6^. In these RCTs, the study populations were undergoing major invasive surgery to the abdomen (for example, laparotomy). These study populations are not comparable to hand trauma patients and so the results are not generalizable. The availability, existing RCT data and presence of national guidelines concerning antimicrobial sutures makes them a logical intervention to evaluate in hand trauma. In order to test antimicrobial sutures in hand trauma surgery, feasibility data on recruitment, compliance and retention must be determined.

## Methods

The Hand And Wrist: AntImicrobials and Infection (HAWAII) feasibility study was designed as prospective, multicentre, randomized, controlled feasibility trial. The study was conducted at three NHS hospitals in the UK. HAWAII was reviewed by the National Research Ethics Service Committee (21/SC/0334) and was carried out in compliance with the Helsinki Declaration. It is registered with the International Standard Randomized Controlled Trial Number Register (ISRCTN10771059). A trainee collaborative model was employed, whereby trainees could collect ‘HAWAII points’ by recruiting, randomizing and completing baseline case report forms (CRFs). Pre-trial audit data indicated that the total number of hand trauma patients attending each site for surgery per month was as follows: 110 *versus* 59 *versus* 60. Progression criteria were generated *a priori* based on these audit data. Meeting green criteria was considered a measure of sufficient feasibility for a definitive trial. Amber criteria triggered a review of study processes by the trial steering committee. Red criteria indicated that a definitive study may not be feasible (*Appendix S1*). A full protocol was published and is available (open access)^7^. This study is reported in accordance with the CONSORT 2010 statement: extension to randomized pilot and feasibility trials (*Appendix S2*)^8^.

### Screening and recruitment

Patients aged 18 years or older with any hand or wrist injury requiring an operation that included the use of sutures were eligible for inclusion. Adults who were unable to give informed consent, who were allergic to Triclosan or who were unable to complete study procedures, including the completion of a patient questionnaire in English, were excluded. Patients with infected wounds, wounds that could not be closed primarily with sutures or with finger nailbed injuries were also excluded. Potentially eligible patients were screened and approached in the Emergency Department (ED), hand trauma clinics or preoperative assessment areas. Once eligibility was confirmed, the study information was distributed and informed consent was attained (*Appendix S3*, *S4*).

### Interventions

The intervention arm received antimicrobial sutures produced by Ethicon (for example, Monocryl Plus, PDS Plus). Antimicrobial sutures could be deployed alone or in combination with standard sutures, and for any part of the operation (for example, deep structural repair, skin closure). The control arm received standard, non-antimicrobial sutures of any kind as per standard practice at each site. Participants received usual pre-, peri- and postoperative care according to site routine practice. Standard care usually involves basic initial wound care (wound washout, application of a dressing, elevation in a sling, tetanus prophylaxis). Prophylactic antibiotics are often prescribed perioperatively, although this can be variable. Usual care data were recorded to inform feasibility of future antimicrobial studies in hand trauma and no co-interventions were prohibited.

### Treatment allocation

Treatment was allocated using a secure, centralized, 24-hour online randomization service. Randomization was performed on a 1:1 basis, stratified by age of the patient and whether the injury was open or closed.

### Blinding

Participants were blinded to the allocation of treatment. Outcome measurement was completed remotely and electronically by the participants. Operating surgeons were aware of the allocation by necessity but were not involved in patient follow-up.

### Outcome measures

#### Primary outcomes

The primary outcomes for this study were measures of feasibility that can inform recruitment, compliance, and retention for a definitive study:

- number of eligible participants;
- number of participants who consent to be included in the study;
- number of eligible participants who are randomized to either the intervention or control;
- number of participants with completed patient-reported outcome measures (PROMs) at the set time points:
  - SSI recorded at 30 days;
  - SSI recorded at 90 days;
  - PROMs completed at 30 days;
  - PROMs completed at 90 days; and
  - PROMs completed at 6 months
- number of participants who develop a site-reported complication, including SSI.

#### Secondary outcomes

##### Surgical site infection

The Bluebelle Wound Healing Questionnaire (BWHQ) was used to detect occurrence of SSI at 30 and 90 days. It maps to the Centers for Diseases Control (CDC) definition of SSI and has been validated for use in UK populations and for completion by participant or observer^9^. The BWHQ was deployed remotely, electronically via REDCap at 30 and 90 days.

##### Hand and wrist function

Two PROMs were deployed to measure hand and wrist function: the Patient Evaluation Measure (PEM) Part 2 and the Patient-Reported Outcomes Measurement Information System: Upper Extremity (PROMIS UE)^10–12^. The PEM consists of 10 questions, addressing different aspects of hand function^10^. PROMIS UE can be administered via a computer adaptive test (CAT), where participant responses guide the system’s choice of subsequent items from the full item bank^11,12^. Both PROMs were deployed remotely, electronically via REDCap at 30 days, 90 days and at 6 months.

##### Health-Related quality of life and return to work

Health-related quality of life (HRQoL) was measured using the EQ-5D-5L, administered at baseline, 30 days, 90 days and 6 months remotely, electronically via REDCap to capture changes in HRQoL^13–15^. Employment status was assessed at 6 months only also *via* REDCap.

### Power and sample size

A sample size of 116 was required to determine acceptable 95 per cent c.i.s for participant compliance and retention, providing usable estimates for a definitive study. The 95 per cent c.i. (Wilson’s method) has a maximum c.i. width of 0.18 given 116 participants.

### Statistical analysis

Descriptive summaries of the data were generated and displayed according to treatment group. The number of eligible participants in total, the number of participants who consented for inclusion and the number of eligible participants randomized were reported descriptively as proportions with 95 per cent c.i.s. Full trial outcome data were reported descriptively. For continuous data, the means and s.d. were calculated with 95 per cent c.i.s and for categorical data, proportions as percentages with 95 per cent c.i.s. A full statistical analysis plan was developed *a priori*.

### Patient and public involvement

The UK Trauma Patient and Public Involvement (PPI) Group helped design the HAWAII study. From this larger group, six individuals were recruited for a project-specific PPI group session to help shape the study. Two PPI formed part of the overall research steering group, both with experience of hand trauma.

## Results

### Demographics

**Fig. 1:**
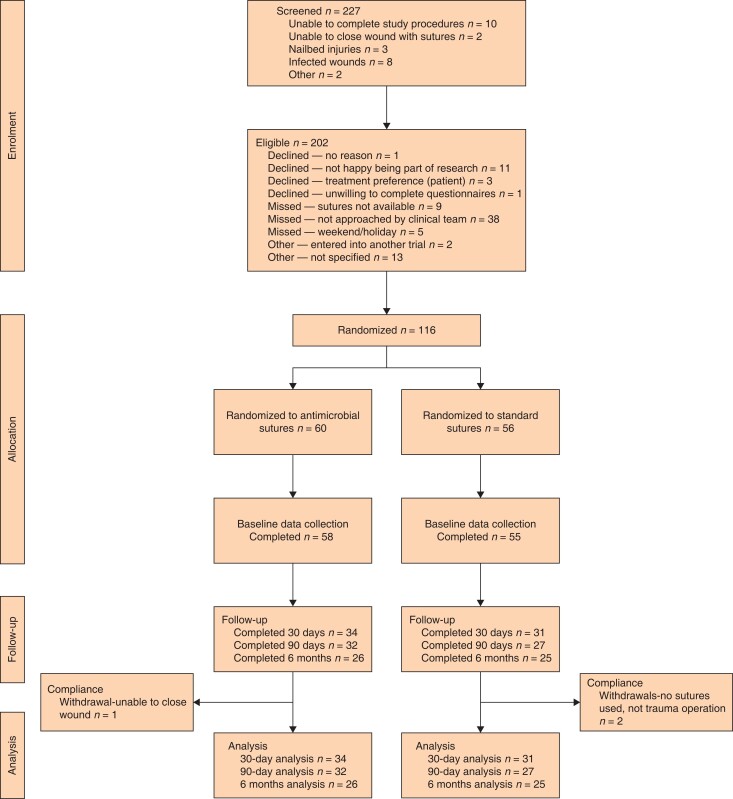
Consort flow diagram

From 10 March 2022 to 2 November 2022, 116 participants were randomly assigned to receive either antimicrobial sutures (*n* = 60) or standard sutures (*n* = 56) alone (*Fig. 1*). Follow-up for the primary outcome was completed in January 2023 and final follow-up was completed in May 2023. Demographics and injury characteristics were comparable at baseline (*Table 1*). The population was predominantly men in their forties. There were more smokers in the control group (31.3 per cent *versus* 16.7 per cent). There were few patients with diabetes or on immunosuppressive therapy in both groups and participants were just above normal BMI on average. Most worked full-time (*n* = 67, 57.8 per cent) and most needed to use their hands for their work (*n* = 78, 67.2 per cent).

**Table 1:** Characteristics of participants

	Antimicrobial sutures	Standard sutures
	*n* = 60	*n* = 56
**Demographics**		
Mean age (s.d.)	44.8 (17.5)	47.2 (20.0)
Male sex (%)	46 (76.7)	36 (64.3)
Smoker (%)	9 (16.7)	15 (31.3)
Diabetes (%)	4 (7.4)	2 (4.2)
Immunosuppression (%)	1 (1.9)	2 (4.2)
Height (s.d.)	174.3 (9.3)	174 (10.6)
Weight (s.d.)	80.0 (17.7)	80 (17.1)
BMI (s.d.)	26.3 (5.2)	26.4 (4.8)
**Employment**		
Working full-time (%)	40 (74.1)	27 (56.3)
Working part-time (%)	4 (7.4)	4 (8.3)
Not working (%)	10 (18.5)	17 (35.4)
Work requiring use of hands (%)	43 (79.6)	35 (72.9)
Self-employed (%)	14 (25.9)	13 (27.1)
**Residential status**		
Own home (%)	50 (92.6)	44 (93.6)
Other (%)	4 (7.4)	3 (6.4)

### Injuries

Injuries were most commonly sharp lacerations or resulting from falls in both groups (*Table 2*). Hands were more commonly injured than wrists, usually the non-dominant limb. Injured structures were comparable between the two groups, with the majority of injuries open and contaminated. Closed injuries accounted for approximately 30 per cent in both groups.

**Table 2:** Characteristics of hand and wrist injury

	Antimicrobial sutures	Standard sutures
	*n* = 60	*n* = 56
**Injury type and mechanism**		
Blunt (%)	3 (5.6)	6 (12.5)
Sharp (%)	29 (53.7)	24 (50)
Crush (%)	8 (14.8)	3 (6.3)
Fall (%)	10 (18.5)	10 (20.8)
Sport (%)	6 (11.1)	3 (6.3)
Confrontation (%)	0 (0)	0 (0)
Other (%)	1 (1.9)	4 (8.3)
Missing (%)	6 (10)	7 (12.7)
**Injury site**		
Hand (%)	45 (76.3)	42 (76.4)
Wrist (%)	13 (22)	13 (23.6)
Both (%)	1 (1.7)	0 (0)
Dominant limb (%)	27 (45.8)	24 (43.6)
Missing (%)	1 (1.7)	1 (1.8)
**Injured structures**		
Skin (%)	38 (64.4)	38 (69.1)
Tendon (%)	15 (25.4)	15 (27.3)
Nerve (%)	12 (20.3)	10 (18.2)
Artery (%)	5 (8.5)	2 (3.6)
Muscle (%)	8 (13.6)	2 (3.6)
Ligament (%)	2 (3.4)	3 (5.5)
Joint (%)	2 (3.4)	3 (5.5)
Bone (%)	25 (42.4)	21 (38.2)
Missing (%)	1 (1.7)	1 (1.8)
**Injury type**		
Open (%)	40 (67.8)	38 (69.1)
Contaminated (%)	36 (61)	36 (65)
Dirty (%)	4 (6.7)	2 (3.6)
Closed (%)	19 (32.2)	17 (30.9)
Missing (%)	1 (1.7)	1 (1.8)
**Destination**		
Admitted (%)	7 (11.9)	8 (14.5)
Discharged TCI (%)	52 (88.1)	47 (85.5)

TCI—‘to come in’.

### Surgical management

Operative management was comparable between groups, with predominantly registrar-led procedures occurring in main theatres or minor operating settings (*Table 3*). Over 80 per cent of procedures were performed under local anaesthetic in both groups. Alcoholic chlorhexidine with full sterile prep and drape was usually employed. The surgical procedures performed reflected the injury types, with most patients undergoing debridement and washout, followed by either tendon or nerve repair and/or open reduction and internal fixation (ORIF). All participants allocated to the intervention group received antimicrobial sutures (100 per cent adherenece, 83.0 per cent for skin closure, 18.6 per cent structural repairs). One participant in the control group erroneously received antimicrobial sutures (1 per cent cross-over). Standard non-adherent dressings were used to protect the surgical wound in the majority of cases. Antimicrobial dressings were used rarely (*n* = 6, 5.2 per cent).

**Table 3:** Operative management

	Antimicrobial sutures	Standard sutures
	*n* = 59	*n* = 55
**Grade of surgeon**		
Consultant (%)	16 (27.1)	14 (25.5)
Associate specialist (%)	8 (13.6)	8 (14.5)
Registrar (%)	28 (47.5)	26 (47.3)
CT/SHO (%)	6 (10.2)	6 (10.9)
ANP (%)	1 (1.7)	1 (1.8)
**Setting**		
Main theatres (%)	39 (66.1)	38 (69.1)
MOPS (%)	18 (30.5)	17 (30.9)
Clinic (%)	1 (1.7)	0 (0)
ED (%)	1 (1.7)	0 (0)
Anaesthetic		
GA (%)	14 (23.3)	10 (18.2)
LA (%)	49 (81.7)	45 (81.8)
Other (%)	1 (1.7)	3 (5.5)
**Surgical preparation fluid**		
Alcoholic chlorhexidine (%)	33 (56.9)	40 (72.7)
Alcoholic betadine (%)	8 (13.8)	3 (5.5)
Aqueous chlorhexidine (%)	11 (19)	8 (14.5)
Aqueous betadine (%)	0 (0)	2 (3.6)
Other (%)	6 (10.3)	2 (3.6)
**Sterile field preparation**		
Full sterile draping (%)	36 (62.1)	35 (63.6)
Aperture drape (%)	21 (36.2)	13 (34.5)
None (%)	1 (1.7)	0 (0)
Other (%)	0 (0)	1 (1.8)
**Operative details**		
Debridement (%)	34 (57.6)	34 (61.8)
Washout (%)	45 (76.3)	42 (76.4)
Joint washout (%)	5 (8.5)	6 (11.1)
Nerve repair (%)	12 (20.3)	4 (7.4)
Tendon repair (%)	12 (20.3)	12 (22.2)
Artery repair (%)	0 (0)	1 (1.9)
MUA (%)	1 (1.7)	1 (1.9)
ORIF (%)	18 (30.5)	14 (25.9)
Open reduction K-wire (%)	2 (3.4)	3 (5.6)
External fixation (%)	0 (0)	0 (0)
**Repair of internal structures**		
Suture repair of internal structures (%)	25 (42.4)	27 (49.1)
Antimicrobial sutures (%)	11 (18.6)	0 (0)
Standard sutures (%)	14 (23.7)	27 (100)
**Repair of skin**		
Suture repair of skin (%)	59 (100)	54 (98.2)
Antimicrobial sutures (%)	49 (83.0)	0 (0)
Standard sutures (%)	8 (23.7)	52 (95.0)
**Wound dressing**		
Standard non-adherent (%)	46 (85.7)	49 (92.5)
Antimicrobial (%)	5 (8.9)	1 (1.9)
Other (%)	3 (5.4)	3 (5.7)
**Splint**		
Plaster of Paris cast (%)	26 (44)	22 (40)
Bandage (%)	0 (0)	2 (3.6)
Zimmer splint (%)	2 (3.3)	3 (5.4)
Other (%)	1 (1.7)	0 (0)
None (%)	30 (53.6)	0 (0)
**Sling**		
High-arm sling (%)	27 (45.8)	21 (38.2)
Other (%)	18 (30.5)	21 (38.2)
None (%)	14 (23.7)	13 (23.6)

ANP, Advanced Nurse Practitioner; CT/SHO, Core Trainee/Senior House Officer; MOPS, minor operating theatres; LA, local anaesthetic; GA, general anaesthetic; MUA, manipulation under anaesthesia.

### Antibiotic management

In the intervention group, 36 participants received preoperative antibiotics (60.0 per cent) and 33 (59.0 per cent) in the control group (*Table 4*). Over 40 per cent of patients in both groups (*n* = 50 total), including open soft tissue injuries and open bony injuries, received preoperative oral antibiotics in the ED, most commonly amoxicillin with clavulanic acid (that is, co-amoxiclav). A further 19 patients received oral antibiotics in the hand trauma clinic, also usually co-amoxiclav. Induction antibiotics were used in a third of cases, some in addition to those already commenced on antibiotics preoperatively. A small proportion of participants received induction antibiotics without preoperative antibiotics: 12 (20.0 per cent) in the intervention group and 13 (23.2 per cent) in the control group. Postoperative antibiotics were prescribed in 19 (17.5 per cent) participants in the intervention group and 9 (16.4 per cent) in the control group. The remainder had already been prescribed antibiotics, or antibiotics were not indicated as judged by the treating clinicians.

**Table 4:** Antibiotic treatment

	Antimicrobial sutures (%)	Standard sutures (%)
	*n* = 60	*n* = 56
**Emergency Department antibiotics**		
Yes (%)	25 (41.7)	25 (45.5)
Co-amoxiclav (%)	21 (35.0)	23 (41.0)
Flucloxacillin (%)	2 (3.3)	1 (1.8)
Clindamycin (%)	0 (0)	0 (0)
Other (%)	1 (1.6)	0 (0)
No (%)	34 (56.7)	29 (52.7)
Unknown (%)	1 (1.6)	1 (1.8)
**Hand trauma clinic antibiotics**		
Yes (%)	11 (18.3)	8 (14.3)
Co-amoxiclav (%)	9 (15.0)	7 (12.5)
Flucloxacillin (%)	1 (1.6)	1 (1.8)
Clindamycin (%)	0 (0)	0 (0)
Other (%)	1 (1.6)	0 (0)
No (%)	48 (80.0)	45 (80.3)
Unknown (%)	0 (0)	2 (3.6)
**Induction antibiotics**		
Yes (%)	21 (35.6)	19 (34.5)
Co-amoxiclav (%)	6 (10.2)	5 (9.0
Flucloxacillin (%)	8 (13.6)	7 (12.7)
Other (%)	7 (11.9)	7 (12.7)
No (%)	31 (52.5)	35 (63.6)
Unknown (%)	7 (11.9)	1 (1.8)
**Postoperative antibiotics**		
Yes (%)	19 (17.5)	9 (16.4)
Co-amoxiclav (%)	8 (13.6)	8 (14.5)
Flucloxacillin (%)	0 (0)	1 (1.8)
Other (%)	1 (1.6)	0 (0)
No—already prescribed (%)	23 (40.4)	20 (36.4)
No—not indicated (%)	24 (42.1)	26 (47.3)
Received prophylactic antibiotics (%)	36 (60.0)	32 (58.2)
Received induction antibiotics only (%)	20 (33.3)	19 (33.9)
Received antibiotics (%)	56 (93.3)	51 (91.1)

### Feasibility outcomes

#### Recruitment

**Fig. 2:**
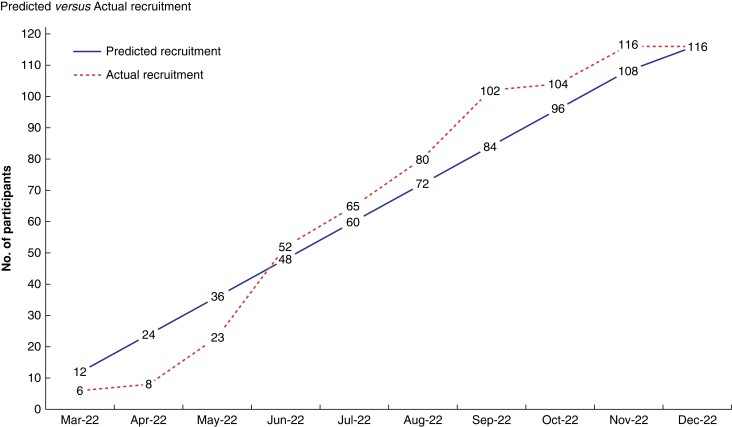
Recruitment graph

In total, 227 patients were screened for eligibility (*Table 5*). Of these, 116 (51.1 per cent, c.i. [44.4–57.8]) were recruited and randomized over 7 months and 23 days (237 days), equating to one randomization every 2 days (*Fig 2*). Screening varied by site: 130 *versus* 64 *versus* 33, with a mean total screening of 75.7 (c.i. [69.2–82.2]) patients per site. The proportion of those screened in terms of the average number of hand trauma surgery patients by site (*a priori* audit data) was: 28.9 per cent (*n* = 130), 7.8 per cent (*n* = 64) and 7.5 per cent (*n* = 33). Recruitment and randomization also varied by site: 67 *versus* 26 *versus* 23, with a mean total recruitment of 38.7 (c.i. [34.2–43.2]) participants per site. Of 227 screened, 202 (89.5 per cent, c.i. [84.2–92.7]) were potentially eligible for inclusion. Of these, 137 (67.8 per cent, c.i. [60.9–74.2]) patients were approached for recruitment with 116 (84.7 per cent, c.i. [77.5–90.3]) being recruited into the trial. Participants were recruited by research nurses, consultants and trainees at all three sites. Each site had a designated Primary Investigator (PI) and at least one NIHR Associate PI. The PIs were all consultant hand surgeons. At one site, two consultants were appointed as NIHR Associate PIs. Another site had a surgical trainee as the NIHR Associate PI and another had an advanced clinical practitioner as the NIHR Associate PI.

**Table 5:** Feasibility outcomes

		*n*	%	c.i. lower	c.i. upper
Screened		227	100	–	–
Randomized		116	51.1	44.4	57.8
Baseline data		114	98.3	39.9	99.8
Pre-injury PROMs		112	96.6	31.4	99.1
Post-injury PROMs		112	96.6	31.4	99.1
Operation data		114	98.3	39.9	99.8
**BWHQ**					
30 days		65	57.5	47.9	66.8
90 days		59	52.2	42.6	61.7
**Hand function PROMs**					
30 days		65	57.5	47.9	66.8
90 days		59	52.2	42.6	61.7
6 months		51	45.1	35.8	54.8
Complications		4	3.5	1.0	8.8
Wound infection		3	2.7	0.5	7.6
Wound dehiscence		1	0.9	0.2	4.8

#### Compliance

Baseline data collection was completed for 114 participants (98.3 per cent, c.i. [94.0–99.8]). Pre- and post-injury PROMs were completed by 112 participants (96.6 per cent, c.i. [91.4–99.1]). Operative data were completed for 114 participants (98.3 per cent, c.i. [94.0–99.8]). Almost all participants received their assigned intervention, with one cross-over from standard sutures to antimicrobial. Three patients were withdrawn (2.6 per cent, c.i. [0.5–7.4]), two as sutures were not used in surgery and one underwent an elective operation (carpal tunnel decompression). There were three complications reported by sites: three incidences of SSI (2.7 per cent, c.i. [0.5–7.6]) and one of wound dehiscence (0.9 per cent, c.i. [0.0–4.7]). There was one serious adverse event (SAE) and no deaths. The SAE related to a participant who sustained a secondary injury from their wound dressing becoming caught in machinery and resulting in a digital amputation.

#### Retention

At 30 days, 65 participants (57.5 per cent, c.i. [47.9–66.8]) completed PROMs, including the BWHQ, PEM, PROMIS UE and EQ-5D-5L. If participants did complete their PROMs, then they completed all questionnaires. At 90 days, 59 participants (52.2 per cent, c.i. [42.6–61.7]) had completed PROMs. At 6 months and final follow-up, 51 participants (45.1 per cent, c.i. [35.8–54.8]) had responded. Follow-up varied across sites at 30 days, where completion rates were 34.6 per cent *versus* 59.1 per cent *versus* 66.7 per cent. At 90 days there was less variation: 50.0 per cent *versus* 52.3 per cent *versus* 54.6 per cent. By 6 months, completion was similar across sites at 42.3 per cent *versus* 46.1 per cent *versus* 46.5 per cent.

### Full trial outcomes

#### Surgical site infection

At 30 days, the mean (s.d.) BWHQ score was 6.4 (5.4) in the intervention group compared to 5.0 (3.7) in the control group (*Table 6*). Using a conservative threshold score of 8 points to determine presence of SSI, eight participants in the intervention group met or exceeded this score (22.9 per cent, c.i. [10.8–41.2]), compared to seven in the control group (22.6 per cent, c.i. [9.6–41.1]). At 90 days, the intervention group had a mean (s.d.) BWHQ score of 4.6 (3.9) and the control group a mean (s.d.) score of 3.4 (3.0). Eight participants had scores indicating SSI in the intervention group (25.0 per cent, c.i. [11.5–43.4]), compared to just two in the control group (7.4 per cent, c.i. [0.9–24.3]).

**Table 6:** Full trial outcomes—surgical site infection

	Antimicrobial sutures				Standard sutures			
30 day	*n* = 34				*n* = 31			
	Mean	SD	c.i. lower	c.i. upper	Mean	SD	c.i. lower	c.i. upper
BWHQ Score	6.4	5.4	4.5	8.3	5.0	3.7	3.2	6.7

	n	%	c.i. lower	c.i. upper	n	%	c.i. lower	c.i. upper
BWHQ SSI	8	22.9	10.8	41.2	7	22.6	9.6	41.1
Treated for SSI	7	20.6	8.7	37.9	8	25.8	11.9	44.6
Required antibiotics (%)	4	11.4	3.3	27.5	4	12.9	3.6	29.8
Required antibiotics +/− surgery (%)	5	14.7	4.9	31.1	6	19.4	7.4	37.5

90 Day	*n* = 32				*n* = 27			
	Mean	SD	c.i. lower	c.i. upper	Mean	SD	c.i. lower	c.i. upper
BWHQ Score	4.6	3.9	3.2	6	3.4	3.0	2.2	4.6

	*n*	%	c.i. lower	c.i. upper	*n*	%	c.i. lower	c.i. upper
BWHQ SSI (%)	8	25	11.5	43.4	2	7.4	0.9	24.3
Treated for SSI (%)	10	31.3	16.1	50	2	7.4	0.9	24.3
Required antibiotics (%)	5	15.6	5.3	32.8	1	3.7	0.9	18.7
Required antibiotics +/− surgery (%)	8	25	11.5	43.4	2	7.4	0.9	24.3

At 30 days, seven participants in the intervention group required treatment for SSI (20.6 per cent, c.i. [8.7–37.9]), of whom four required antibiotics alone and five required surgical intervention. In the control group, eight participants required treatment for SSI (25.8 per cent, c.i. [11.9–44.6]), four with antibiotics alone and six requiring surgery. By 90 days, 10 participants required treatment for SSI in the intervention group (31.3 per cent, c.i. [16.1–50.0]), of whom 8 required surgical intervention. Only two required treatment in the control group (7.4 per cent, c.i. [0.9–24.3]). There was no signal of a beneficial effect of antimicrobial sutures in terms of SSI, with poorer BWHQ scores in the intervention group and higher proportions of participants requiring treatment for SSI.

#### Hand function, health-related quality of life and employment

**Fig. 3:**
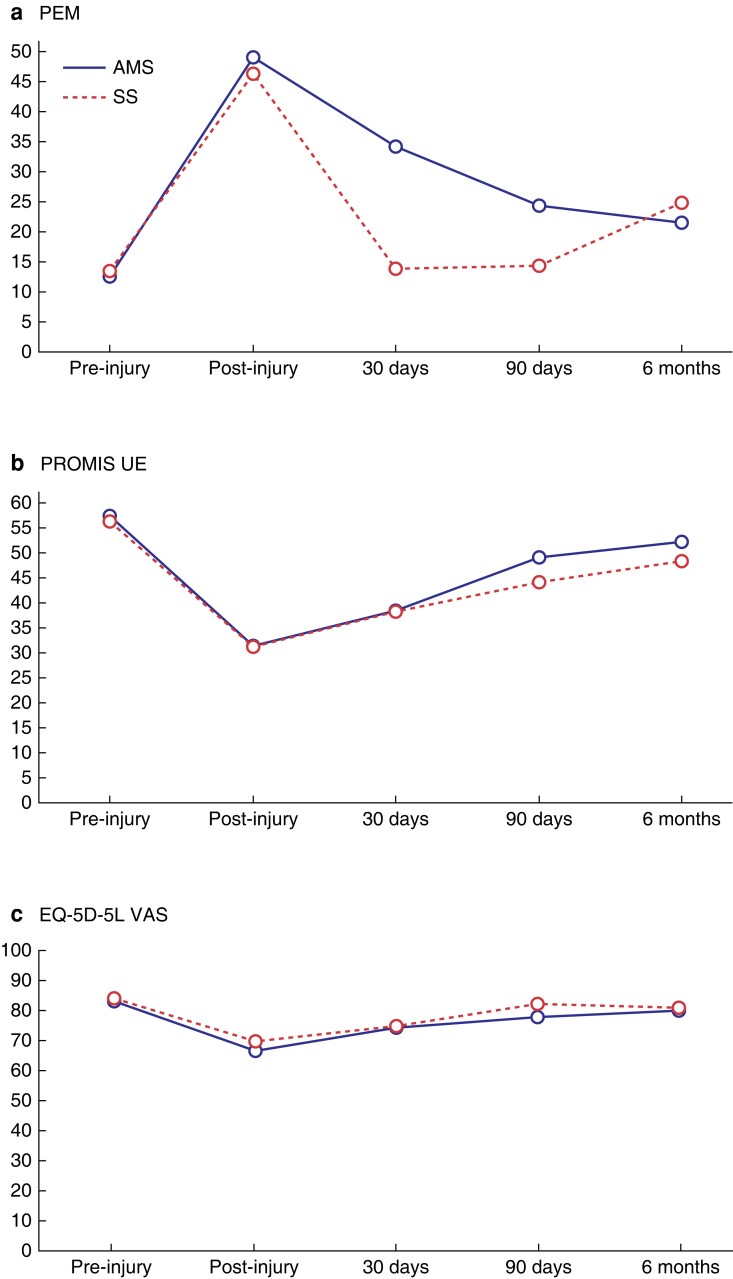
PROM scores at follow-up time points **a** PEM scores at follow-up time points (high score = poor hand function), **b** PROMIS UE scores at follow-up time points (high score = better upper limp function) and **c** EQ-5D-5L VAS scores at follow-up time points (high score = better quality of life).

Similar trends were seen in the PEM and PROMIS UE scores across the study (*Fig. 3a,b*, *Table 7*). Participants in both groups experienced worsening scores from pre-injury to post-injury time points, with subsequent improvement in scores up to final follow-up at 6 months. The EQ-5D-5L scores followed a similar but less marked pattern, with an initial drop followed by recovery over the study period (*Fig. 3c*) By final follow-up, scores across all three measures had not reach pre-injury levels across all outcome measures. There were more self-reported SSIs in the antimicrobial sutures group, paralleled by slightly worse PROMIS UE (48.9 *versus* 44) and EQ-5D-5L VAS scores (77.5 *versus* 81.8) at 90 days compared to the control group. The mean (s.d.) time taken off work was 20.6 (29.4) days in the antimicrobial sutures group *versus*16.5 (30.5) days in the standard sutures group. A small proportion in both groups had to change their type of work as a result of their injury (11.5 per cent, antimicrobial *versus* 8.0 per cent, standard). Similarly to SSI, there was no evidence of beneficial effect in the antimicrobial group, with generally poorer scores across outcome measures.

**Table 7:** Full trial outcomes—hand function and HRQoL

	Antimicrobial sutures				Standard sutures			
	*n* = 60				*n*-56			
		**SD**	**c.i. lower**	**c.i. upper**		**SD**	**c.i. lower**	**c.i. upper**
**Pre-injury**	*n* = 58				*n* = 54			
PEM	12.4	3.6	11.5	13.5	13.4	7.1	11.5	15.3
PROMIS UE	57.3	5.6	55.8	58.8	56.1	5.4	54.6	57.6
EQ-5D-5L index	0.9	0.1	0.9	0.9	0.9	0.2	0.9	1
EQ-5D-5L LSS	5.8	1.8	5.3	6.3	6.2	2.1	5.6	6.8
EQ-5D-5L VAS	83	15.4	82	90.1	83.9	12.4	80.5	87.3
**Post-injury**	*n* = 57				*n* = 55			
PEM	49	13.2	45.5	52.5	46.3	16.2	41.9	50.7
PROMIS UE	31.3	2.7	30.6	32	31.2	2.8	30.4	32
EQ-5D-5L index	0.6	0.2	0.6	0.7	0.6	0.2	0.6	0.7
EQ-5D-5L LSS	10.6	3.1	9.8	11.4	10.6	2.8	9.8	11.4
EQ-5D-5L VAS	66.4	19.2	61.3	71.5	69.6	18.3	64.7	74.6
**30 days**	*n* = 34				*n* = 31			
PEM	34.1	12.4	29.8	38.43	31.7	13.8	26.6	36.8
PROMIS UE	38.3	2.9	37.2	39.3	38.2	3	37.1	39.3
EQ-5D-5L index	0.7	0.2	0.6	0.8	0.7	0.2	0.6	0.8
EQ-5D-5L LSS	8.8	3	7.8	9.8	8.4	2.7	7.4	9.4
EQ-5D-5L VAS	74.1	16.2	68.5	79.8	74.7	18.1	68.1	81.3
**90 days**	*n* = 32				*n* = 27			
PEM	24.3	10	20.7	27.9	26.7	14.2	21.1	32.3
PROMIS UE	48.9	4.2	47.4	50.4	44	3.6	42.6	45.4
EQ-5D-5L index	0.8	0.1	0.8	0.8	0.8	0.2	0.7	0.9
EQ-5D-5L LSS	6.6	1.4	6.1	7.1	7.5	2.2	6.6	8.4
EQ-5D-5L VAS	77.5	18.3	70.9	84.1	81.8	15.4	75.7	87.9
**6 months**	*n* = 26				*n* = 25			
PEM	21.4	10.5	17.2	25.6	24.8	15.6	18.4	31.2
PROMIS UE	52.0	10.5	47.8	56.2	48.2	11.4	43.5	52.9
EQ-5D-5L index	0.9	0.1	0.8	0.9	0.8	0.2	0.7	0.9
EQ-5D-5L LSS	6.2	1.2	5.7	6.7	7.1	2.3	6.2	8.1
EQ-5D-5L VAS	79.9	13.8	74.3	85.5	80.8	14.8	74.7	86.9
Days off work	20.6	29.4	8.7	32.5	16.5	30.5	3.9	29.1
Changed employment	3	11.5	2.5	30.2	2	8.0	1.0	26.0

VAS, visual analogue scale; LSS, level sum score.

#### Harms

No harms in either group arose as a result of treatment allocation.

## Discussion

This feasibility study has demonstrated that the recruitment and randomization of hand trauma patients to antimicrobial sutures is expeditious and achievable. Despite delays in site set-up resulting from the COVID-19 pandemic, we were able to recruit our target number within 8 months, closing the study to recruitment earlier than anticipated. This finding is particularly critical in hand trauma trials, where two NIHR-funded (Health Technology Assessment stream) RCTs have recently been closed due to recruitment issues as part of the ‘Future of UK Clinical Research Delivery: 2022 to 2025 implementation plan’^16–18^. To further improve recruitment in future RCTs of antimicrobials in hand trauma, frameworks such as the Qualitative Research Integrated within Trials (QuinteT) Recruitment Intervention (QRI) and QRI-Two could be employed^19,20^.

Trial activity, including recruitment to HAWAII, was managed *via* WhatsApp, an established methodology in trial coordination, between consultants, research nurses and trainees to ensure potentially eligible patients were approached as often as possible^21^. The online systems for baseline data collection and randomization worked well and there were no system failures throughout the study. We had very high completion rates for baseline and operative data collection across all sites. Intervention fidelity and adherence were excellent. One participant in the control group mistakenly received antimicrobial sutures. In the intervention group, all participants received antimicrobial sutures for either skin closure, structural repair or both. There were no issues of equipoise and no occurrences of participant unblinding.

In terms of progression to a full definitive trial, parameters for screening, recruitment and retention were set in the protocol stage^22,23^. The red criteria for screening and recruitment were met (screen <120 patients per month, recruit <24 participants per month). The amber criteria for retention was met (>50 per cent retention). The green criteria for compliance was met (>80 per cent compliance). In hindsight, this demonstrates that clinical audit data are not a reliable source for setting progression criteria targets.

Retention was the key issue in terms of feasibility. At the primary analysis for a potential full trial, 30 days post surgery, we had only 57.5 per cent completion of remote electronic PROMs by participants. This dropped to 52.2 per cent by 90 days and 45.1 per cent by 6 months. While this is concerning and falls well below the target of >90 per cent or more, it provides useful feasibility information. Using primarily automated, electronic, remote follow-up only, we were able to achieve nearly 60 per cent retention at 30 days. Hand trauma affects a young, predominantly male population and therefore it is realistic to expect difficulties in following up this hard-to-reach population^24,25^.

Best-practice guidance for improving retention in RCTs was published in 2017^26^. This guidance was focused on postal questionnaire responses rather than the contemporary electronic methods employed in HAWAII, although many strategies are applicable. Telephone-based follow-up with reminders is a staple for a trials unit-supported trial and has been successful in achieving better retention in musculoskeletal trials^27^. Monetary incentivization is an additional, potentially useful strategy to improve questionnaire response, supported by reliable evidence^28^. This could be incorporated into a definitive RCT in hand trauma and may be beneficial, considering most common hand trauma occurs in the most deprived sections of society. An incentive valued at £5–£20 was recommended^26^. There was dissent in qualitative analysis as to whether enhanced communication, including repeated reminders, are effective^26^.

The use of validated and efficient questionnaires in HAWAII was recommended by evidence and PPI workshop discussion. We used contemporary PROMs, delivered electronically. For PROMIS UE, we used the CAT version to improve efficiency and reduce questionnaire burden. Since this study, a CAT has been developed for the PEM, which could be incorporated into a future definitive study to reduce burden and improve completion rates, as well as having the added advantage of item response theory, validated, interval scale measurement^29,30^. A combination of strategies should be employed in future trials of antimicrobials in hand trauma. This is an area for future work, focusing on multistakeholder input (for example, interviews) on strategies to improve retention, involving PPI representatives and clinicians.

Despite poor retention, useful follow-up data were attained that can inform a definitive study. All participants who did respond completed all questionnaires sent to them, resulting in complete data for all responders. Three wound infections were reported by sites, all occurring in participants allocated to standard sutures. One occurred in a wound distant to the trial wound but on the same limb. The other two were superficial SSIs requiring oral antibiotics only. Using only these data, the SSI risk in this study would be 2.7 per cent. At 30 days, 65 participants had completed the BWHQ. This includes self-reporting of interventions to treat SSI and so gives a different angle to the site-reported figures, including those that had been managed in primary care. Across both groups, 15 participants reported having received an intervention for SSI by 30 days: 23.1 per cent. Of these, 11 required a surgical intervention, while 8 required antibiotics alone. By 90 days, 59 had completed the BWHQ and 12 had reported receiving interventions for SSI, 20.3 per cent, 10 of whom underwent a physical intervention and 6 had oral antibiotics.

The use of adjunctive antimicrobial interventions, including antibiotics, provides useful feasibility data for future studies in this area. A meta-analysis of antibiotics in hand trauma found no evidence of effect in terms of reducing SSI, although this review was limited by low-quality primary data^31^. Despite the findings of this review, and the previous lack of data on the baseline risk of SSI, systemic antibiotics are routinely used in hand trauma. In HAWAII, over 90 per cent of participants received systemic antibiotics, with 40 per cent receiving antibiotic prophylaxis in the ED. Considering the lack of evidence supporting the effectiveness of this strategy and the commonality of hand trauma, this raises concerns regarding antibiotic stewardship and antimicrobial resistance^32–35^. The use of topical antimicrobial prophylaxis was comparatively rare, with only six participants receiving antimicrobial dressings. As there is no evidence to support the use of antimicrobial dressings in hand trauma, this is somewhat reassuring^36^. Promisingly, the most commonly used surgical preparatory fluid was alcoholic chlorhexidine. Recent research has identified this as the most effective fluid for preventing SSI in hand surgery, although the data pertaining to hand trauma were inconclusive^37–39^. Further work is needed to establish evidence-based guidelines that can be successfully implemented into clinical practice, based on robust cost-effectiveness, health economic and sustainability analyses.

In terms of improving efficiency of a future definitive trial of antimicrobials in hand trauma surgery, the use of linked real-world data to improve follow-up data completion could be advantageous^40^. A 2022 systematic review described data-linked RCTs in surgery, with 2 of the 19 included RCTs using data linkage for outcome data collection^41^. Importantly, the fidelity of the data to which the trial is linked must be considered if this approach is used. In summary, a definitive RCT of antimicrobial sutures in hand trauma surgery may be feasible, as long as retention of study participants to the primary endpoint can be attained. Data from this study can be used to inform future trials of antimicrobial interventions in hand trauma populations. Evidence-based retention strategies, guided by discussion with hand trauma patients and clinical stakeholders, should be considered in future definitive trials.

## HAWAII collaborative

Ana V. Dias, Cara Jenvey, Ciaran Brennan, Haseeb Khawar, Keji Fakoya, Kerry J. Anderson, Mark Williams, Muhammad Haruna, Nizar Ismail, Usman Fuad, Alexander Baldwin, Mohammed Eldolify, Le Yu Mon, Mina Ip, Soma Farag, Hasaan Khan, Simon J. M. Parker, Yasmeen Khan, Kathryn Lewis, Tessa Sewdin, Bobbie Sanghera, Geraldine Hambrook, Eve Fletcher, Suzy Dean.

## Supplementary Material

znad298_Supplementary_Data

## Data Availability

The data generated from this study are not publicly available but may be made available upon specific request to the corresponding author.
