# Supplementary material for: The Hand and Wrist: AntImicrobials and Infection (HAWAII) trial
Source: Br J Surg. 2023 Sep 27;110(12):1774–84. doi: 10.1093/bjs/znad298 (PMC10638545; doi:10.1093/bjs/znad298)
Supplement: znad298_Supplementary_Data [file znad298_supplementary_data.docx]

#

# Appendices

## Appendix 1. Feasibility progression criteria


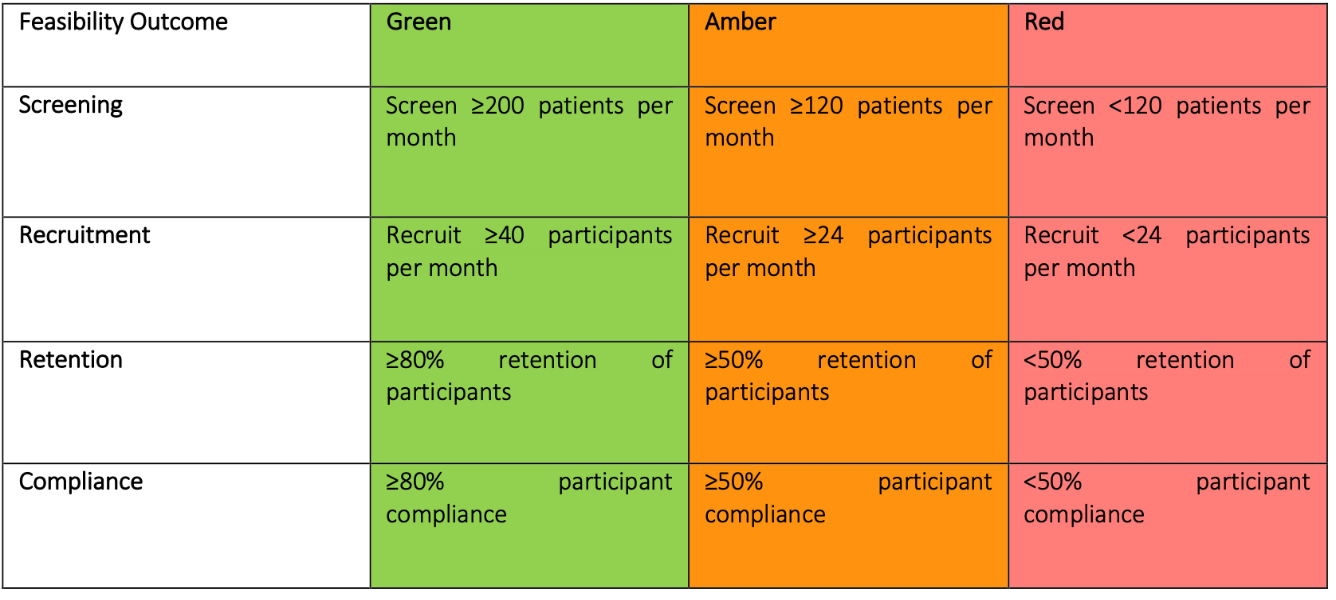


## Appendix 2. CONSORT Checklist: HAWAII Feasibility Study


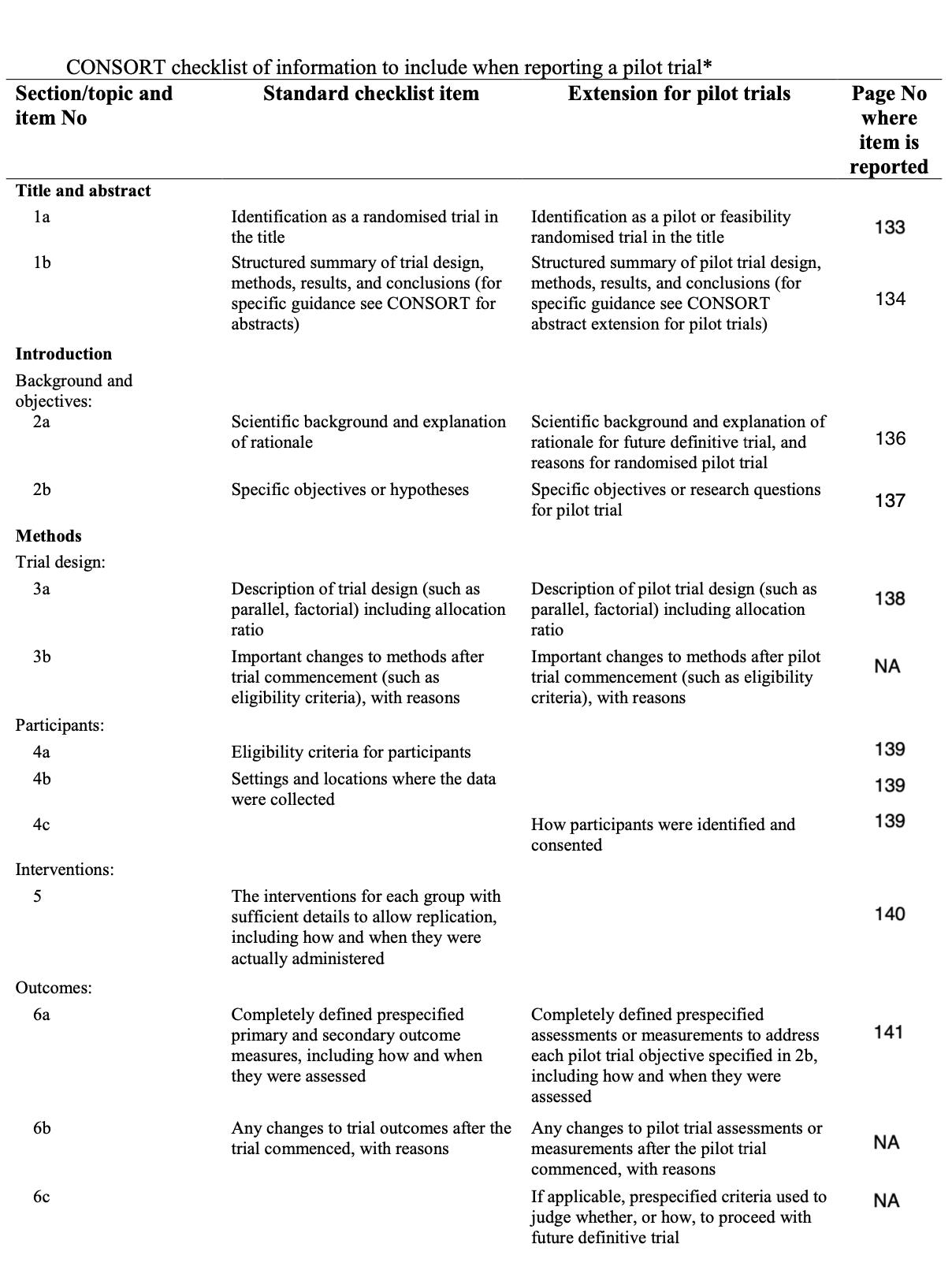


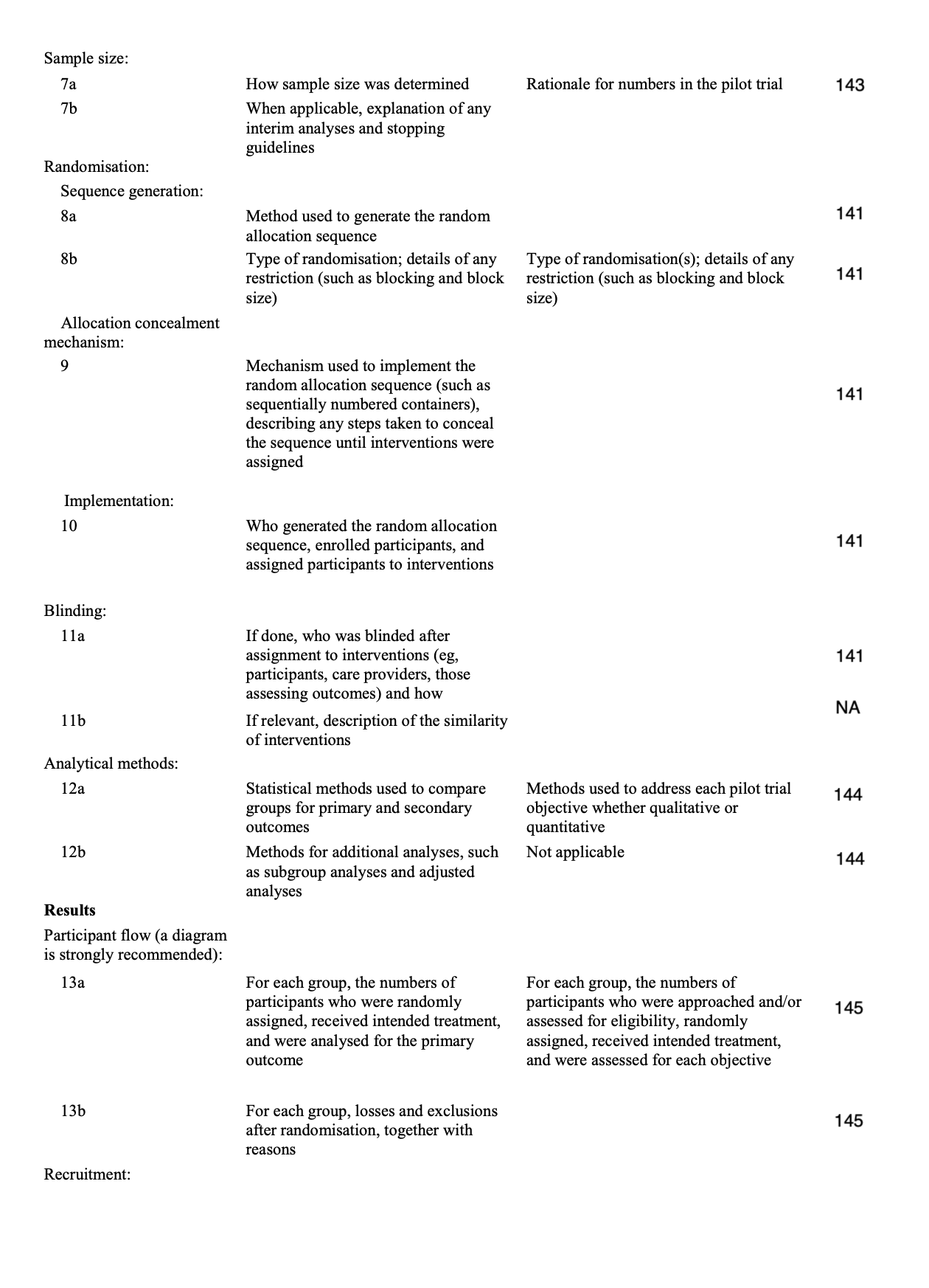


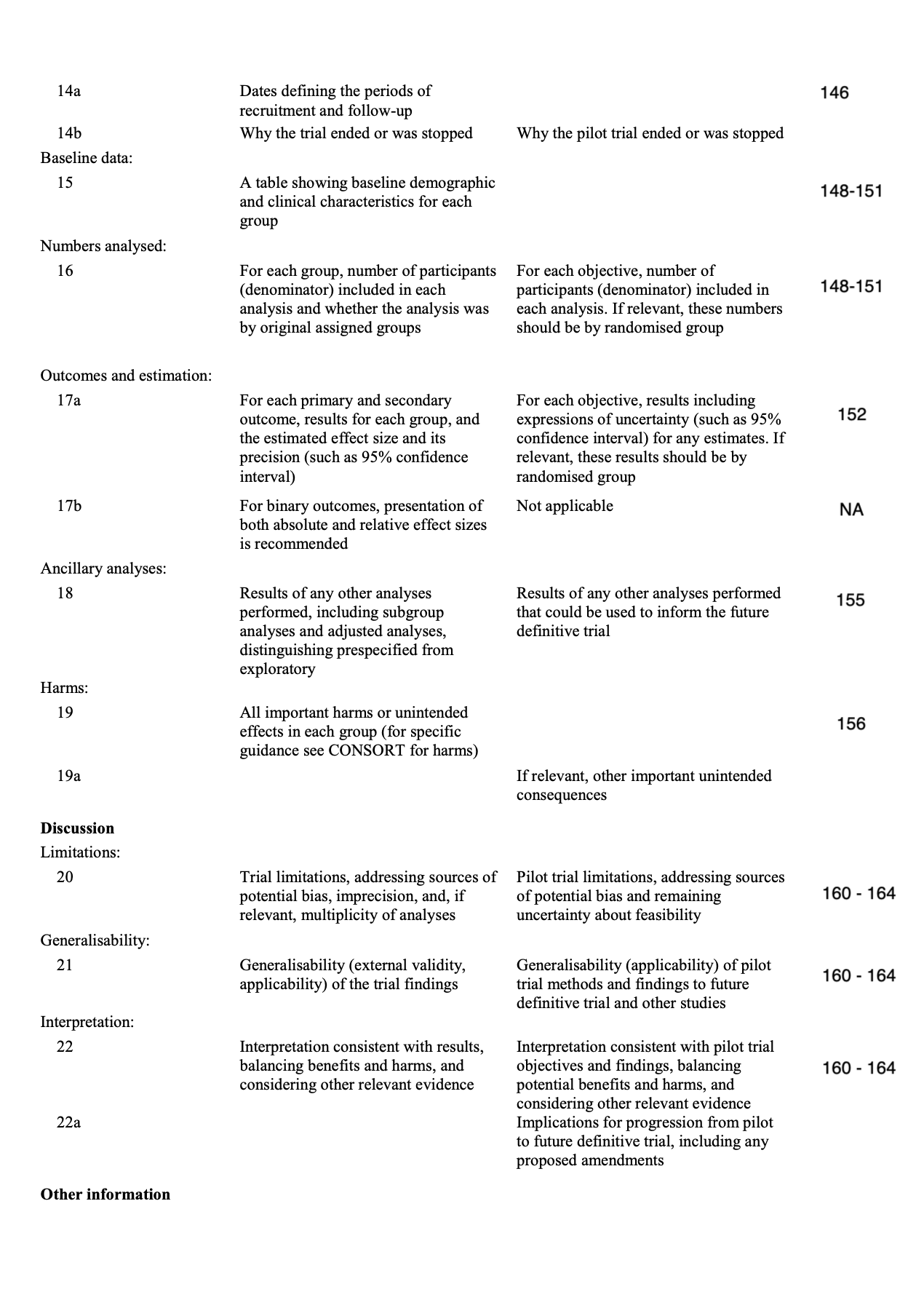


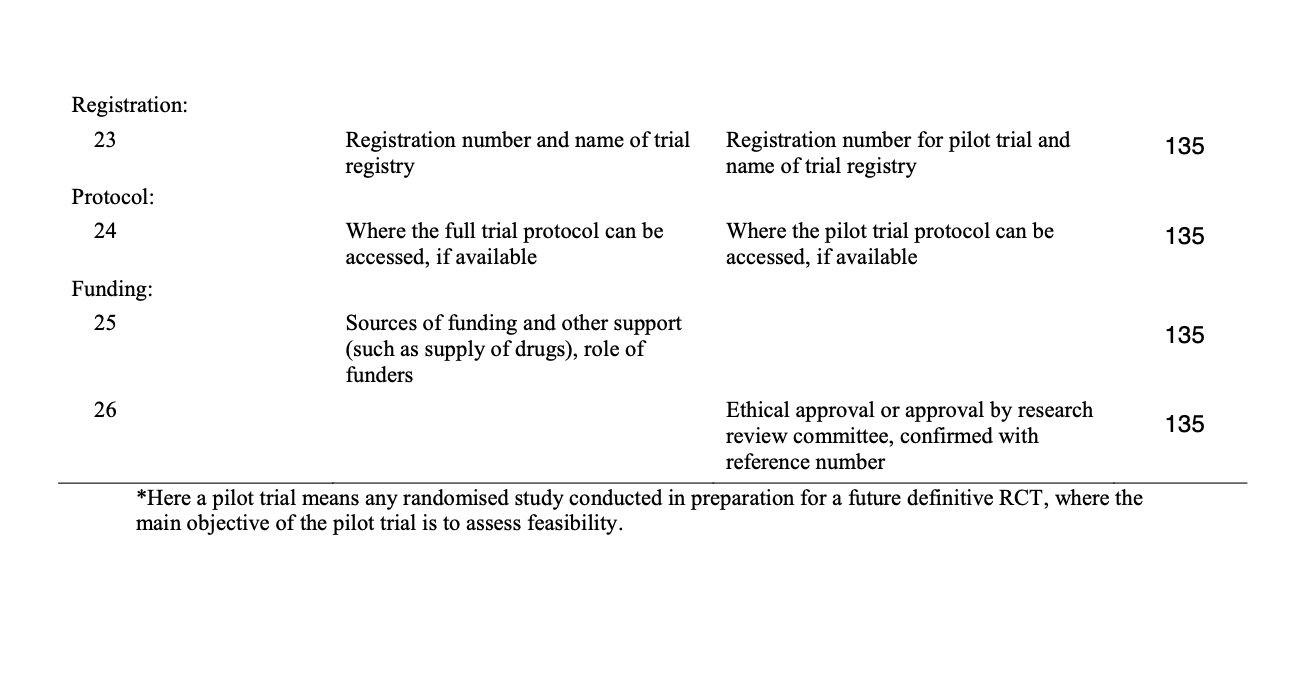


##
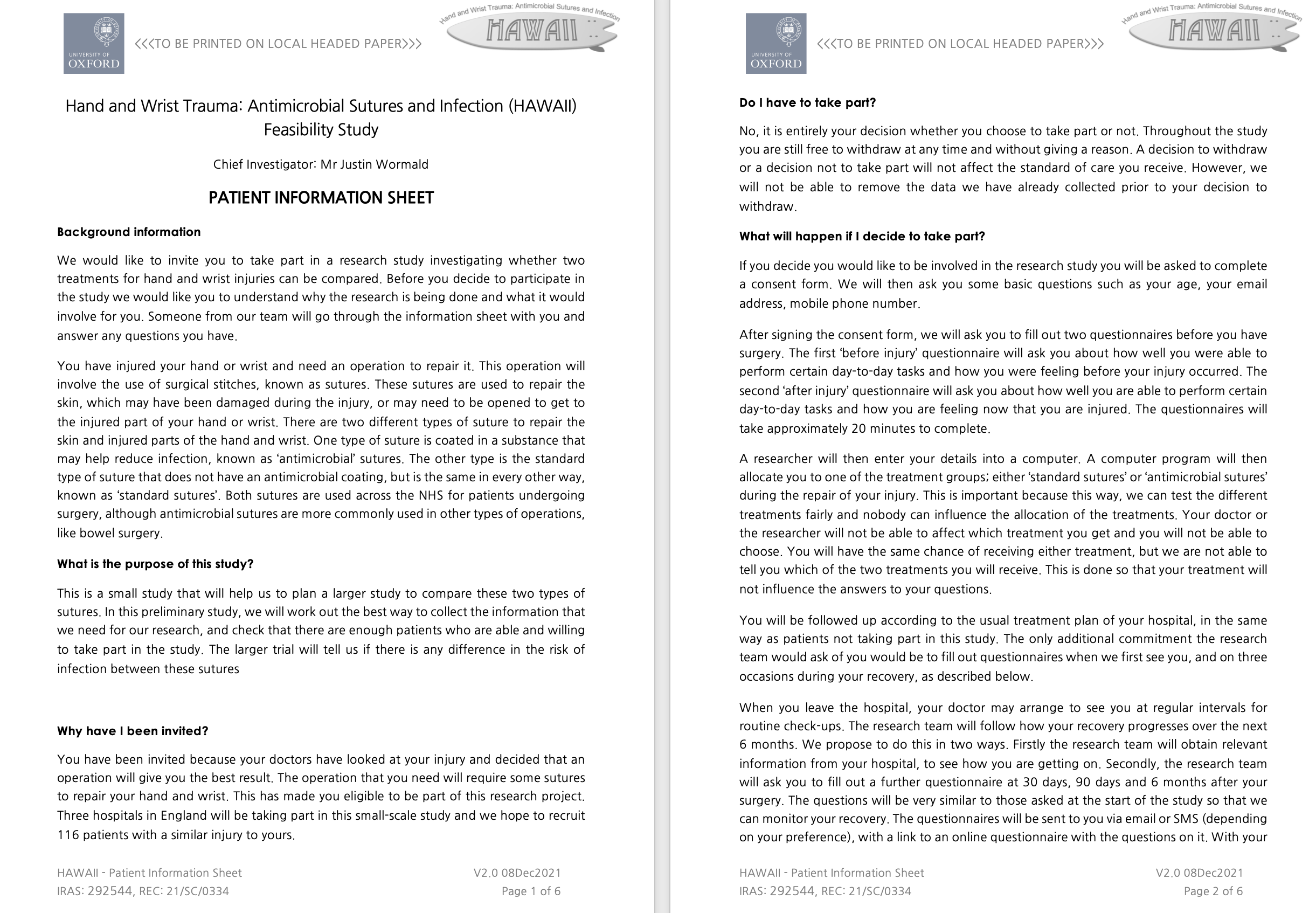
Appendix 3. HAWAII Participant Information Sheet


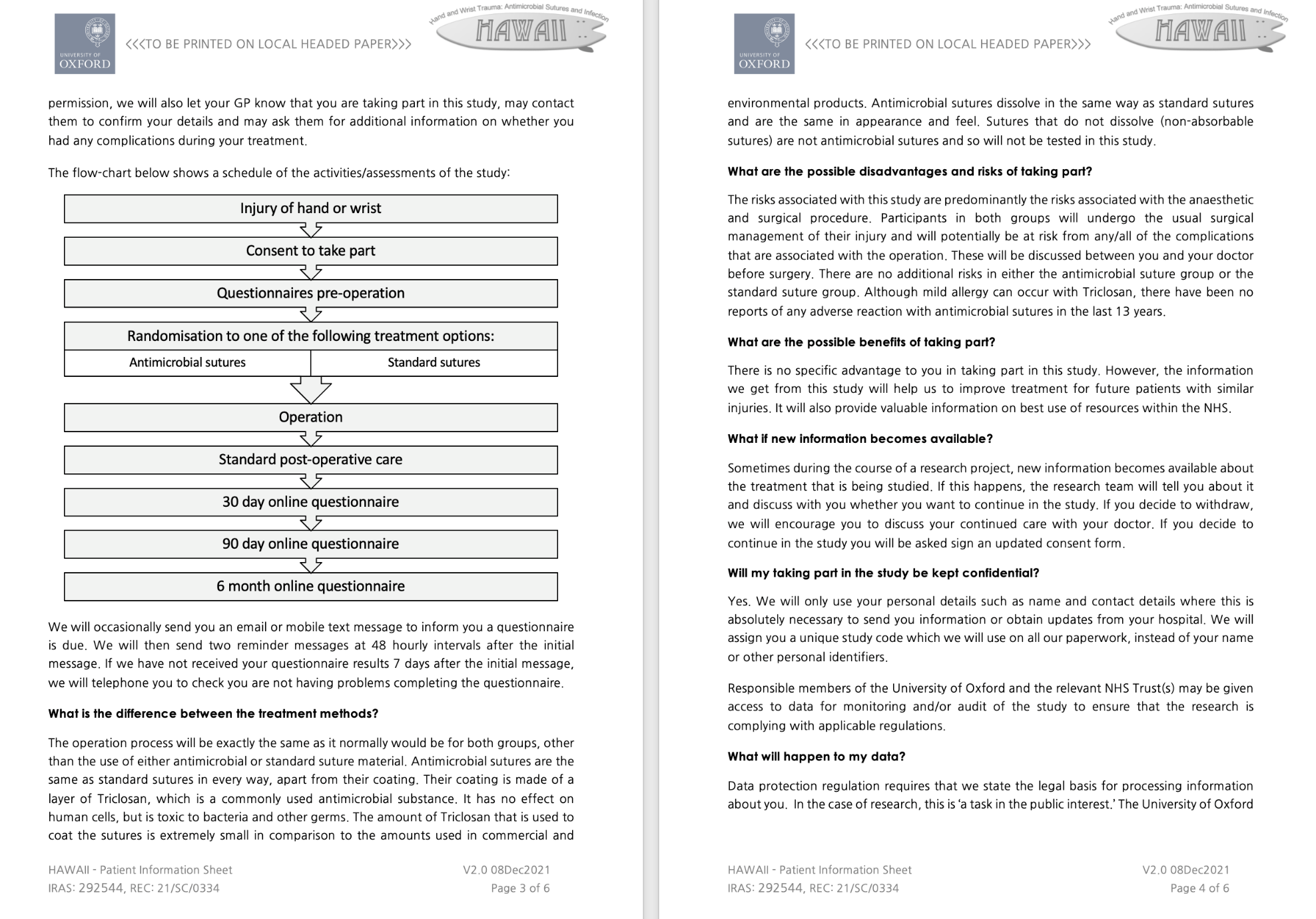

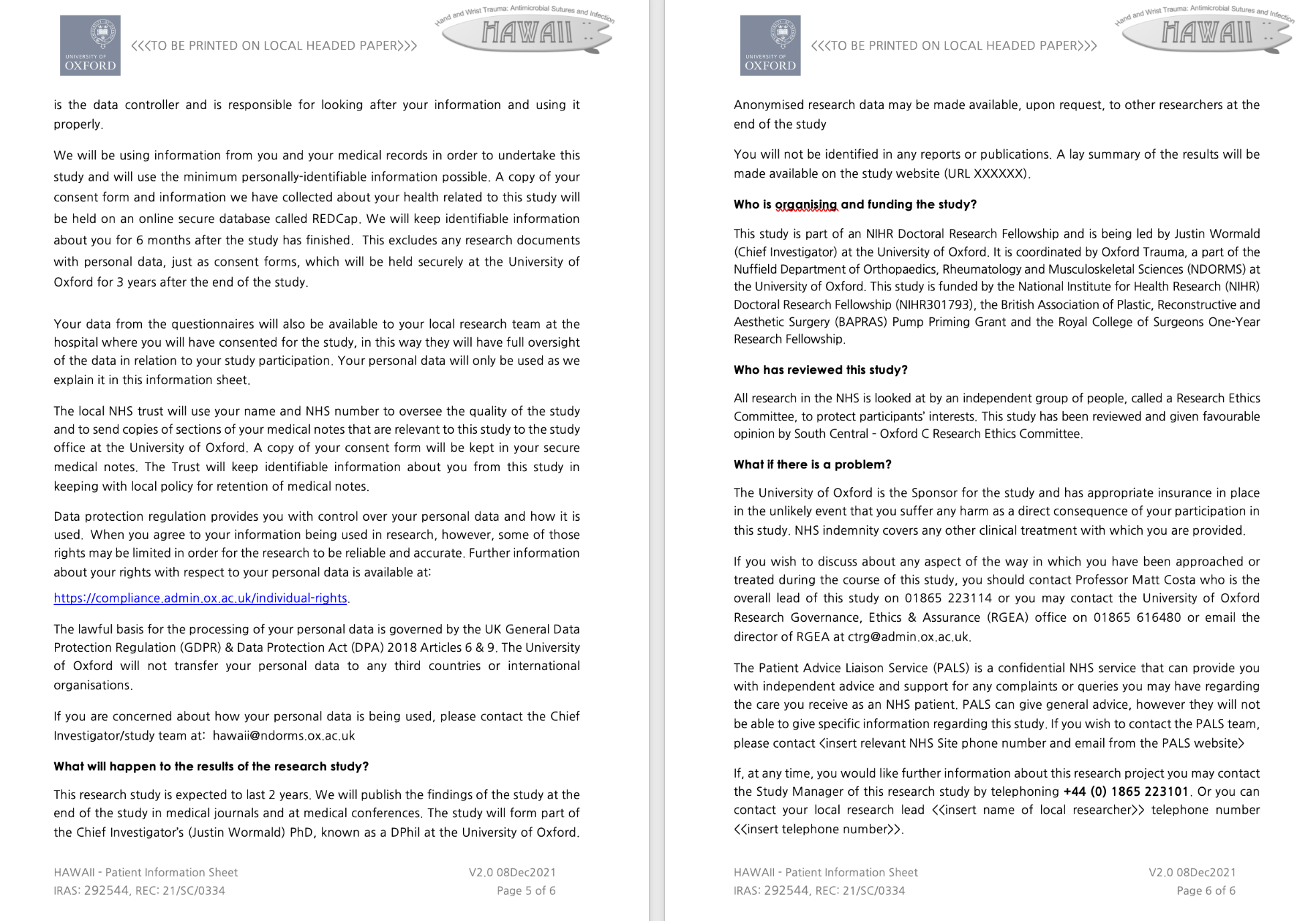


## Appendix 4. HAWAII Consent Form
